# Commentary: ARC: a framework for access, reciprocity and conduct in psychedelic therapies

**DOI:** 10.3389/fpsyg.2023.1248967

**Published:** 2023-12-11

**Authors:** Tahlia R. Harrison

**Affiliations:** Laboratory for Culture and Mental Health Disparities, University of Ottawa, Ottawa, ON, Canada

**Keywords:** bioethics, psychedelic-assisted therapy, culturally responsive and trauma-informed practice, research ethics, psychedelics

## Introduction

The authors introducing the ARC framework for access, reciprocity, and conduct in psychedelic therapies offer a comprehensive and thoughtful methodology for creating a more unified framework of conduct within the realm of psychedelic therapies. As a clinician, bioethicist, and researcher committed to culturally responsive practice, it is encouraging to see more literature exploring ethical and equitable infrastructures for psychedelic care to support the rapid expansion of P-AT in research and clinical settings (Spriggs et al., 2023).

## Discussion

The authors present the Access, Reciprocity, and Conduct (ARC) framework for guiding ethics related to psychedelic-assisted therapy (P-AT). Resting on the foundations of ARC are three pillars dedicated to “representing a commitment to equitable access to psychedelic therapies (Access), a respect for traditional and spiritual uses of psychedelics (Reciprocity), and the safe and ethical delivery of P-AT in clinical settings (Conduct)” (Spriggs et al., 2023). ARC offers the field both a clinical and community-based approach rooted in ethics (Spriggs et al., 2023). Different stakeholders are relevant to each pillar, with ARC aiming to highlight their interdependence within as imperative for robustly supporting the future growth of practice, policy, industry, and research. Background for each of the pillars and the development process for ARC are discussed in detail throughout (Spriggs et al., 2023).

While the authors clearly note that this is a starting point for developing the criteria across stakeholders (Spriggs et al., 2023), future scholarship could benefit by the inclusion of information about the process for choosing representatives from each sector of the psychedelic community. The authors write that “The stakeholders have been identified to be representative of the actors most closely implicated in the ARC pillar in question, and come from research, industry, community, anthropological, policy, and indigenous contexts” (Spriggs et al., 2023). This is helpful insofar as it goes, but how are the authors judging who is most “closely implicated” with P-AT? While most of us are coming from a place of improving the safety of psychedelic therapy space, a few have caused profound harm. This fact makes it imperative that we have complete transparency as to *who* is creating codes of conduct and *how* they are doing so (Harrison, 2023; McNamee et al., 2023). How is each stakeholders' background being reviewed and what measures are being taken to verify that each stakeholder engages in safe and ethical practice?

In my view, any effective approach to governance of P-AT must recognize the importance of collective impacts, that is, those aspects of P-AT that are essential to protecting the physical, psychological, social, political, and environmental wellbeing of vulnerable clinical and research participants (Solomon, 2023). This will require a broader epistemological lens that attends to things like epistemic justice and other ethical issues often ignored in Western bioethics (Pratt and de Vries, 2023). Part of practicing culturally responsive care is looking beyond Eurocentric philosophy (Berger and Miller, 2021). We must also look outward by shifting the focal point from the anthropocentric to the ecocentic: what is good for the collective (including non-human life) is inherently good for humanity. The authors allude to this as part of the value of culturally responsive care; I implore them to define “culturally responsive” at a more granular level. This will help others to understand and implement this framework.

There are many in the field of bioethics advocating for culturally responsive practice [see e.g., Berger and Miller, 2021)] while also recognizing issues of systemic racism and other barriers to implementation of it. Including others committed to these principles from the bioethics community could be a useful addition to the group of stakeholders (if not included already). It's possible many of these questions will be addressed in the follow-up pieces the authors have referenced to be published and I look forward to seeing the continued development of the ARC framework.

## Author contributions

The author confirms being the sole contributor of this work and has approved it for publication.
